# Follower Dependence, Independence, or Interdependence: A Multi-Foci Framework to Unpack the Mystery of Transformational Leadership Effects

**DOI:** 10.3390/ijerph17124534

**Published:** 2020-06-24

**Authors:** Qing Lu, Yonghong Liu, Xu Huang

**Affiliations:** 1 Department of Human Resources Management, Zhongnan University of Economics and Law, Wuhan 430073, China; 2 Department of Management, University of North Carolina at Greensboro, Greensboro, NC 27412, USA; y_liu24@uncg.edu; 3 Department of Management, Hong Kong Baptist University, Kowloon, Hong Kong, China; xuhuang@hkbu.edu.hk

**Keywords:** transformational leadership, leadership process, multiple-mediator model, follower performance

## Abstract

We develop a multi-foci framework—“follower dependence,” “follower independence,” and “leader-follower interdependence”—to explain how transformational leadership influences follower performance. Follower’s personal identification with the leader, psychological empowerment, and leader-member exchange are employed as proxies for each of the three foci. Two separate studies consistently show that personal identification (denoting a “follower dependence” focus) is a more salient mechanism than the other two in explaining the transformational leadership-organizational citizenship behavior relationship. These results suggest transformational leadership is perhaps a theory of follower dependence rather than independence or interdependence. Theoretical implications, limitations, and future directions are discussed.

## 1. Introduction

For decades, organizational scholars have recognized transformational leadership as the most effective form of leadership that stimulates subordinates to sacrifice their self-interest for the sake of organizational goals [1,2]. Transformational leaders induce positive performance outcomes through eliciting a variety of followers’ psychological reactions, including but not limited to increased intrinsic motivation [3], perceptions of justice [4], and collective identity [5]. Nevertheless, research unpacking the underlying processes of transformational leadership seems to have faded in the last few years.

Do we really have sufficient knowledge of the transformational leadership process? Given that recent reviews have consistently suggested that we reflect on this area [6], our answer to this question is “No”. We contend that, although prior transformational leadership research has demonstrated its importance in influencing various followers’ psychological mechanisms, the key mechanism(s) of this theory has/have not been identified, thereby making its explanatory contribution neither clear nor easily identifiable relative to other leadership theories [7]. For example, participative leadership theory [8] and empowering leadership theory [9] specifically emphasize the awakening of follower independence (i.e., their participation and active roles) in the leadership process. By contrast, this feature of transformational leadership theory seems not that clear—our extant understanding seems to suggest that this is a comprehensive leadership theory that not only enables followers’ independence in doing their jobs [10], but also increases followers’ dependence (upon the leader) for recognition and guidance [11] and builds interdependent and long-term relationships with followers [12].

This understanding of transformational leadership theory may reflect the fact that transformational leadership is indeed powerful in soliciting various individuals’ psychological mechanisms. Nevertheless, just as Bono and McNamara [13] suggested, this situation may also be the result of traditional practices of examining the transformational leadership process from one single mechanism (or mediator), making the mediating role of each mechanism unique. To specify the pivotal feature of this leadership theory and avoid the pitfalls of examining one mediator at one time, the purpose of this paper is thus to develop an integrative model that includes all three major mechanisms or “foci” (i.e., “follower dependence [upon the leader],” “follower independence [from the leader],” and “leader–follower interdependence”) that have appeared in prior transformational leadership research. According to Blau [14], these “foci” represent three general postures a leader can have in relation to his/her followers. Specifically, we use followers’ personal identification with the leader to represent the “follower dependence” mechanism because, upon identifying with and internalizing the leader’s values, followers’ work motivation and self-esteem depend highly on the leader’s recognition [11]. We use psychological empowerment to represent the “follower independence” mechanism because this factor denotes follower’s self-initiatives that are possibly nurtured by transformational leaders [15]. Lastly, leader–member exchange (LMX) is used to represent the “leader–follower interdependence” mechanism because of LMX’s emphasis on “the special interdependent relationship of leader and member” [16] and “mutual outcome instrumentalities” [17]. With respect to the outcomes of interest, we focus on in-role task and extra-role performance (organizational citizenship behavior, OCB). Transformational leadership has been argued to motivate followers to perform beyond expectations [1], and the idea of “beyond expectations” can be conceptualized differently. This concept can refer to either directing additional effort toward in-role and required tasks (i.e., task performance) or engaging in discretionary or additional-role work behaviors that are not required by job descriptions (i.e., OCB) [18].

The current study aims to advance our extant understanding of transformational leadership theory via combining the three different mechanisms or “foci” as simultaneous processes and presenting a more complete picture of the inner workings of the transformational leadership process. Our study differs from prior transformational leadership research that either emphasized one single mediator [4,12] or examined multiple mediators in sequence [19,20]. One advantage of our simultaneous mediation approach is that the pivotal mechanism could be demonstrated. Just as Edwards and Berry [21] and Van Der Ven [22] advocated, analyzing several alternative intervening mechanisms can tremendously improve theoretical precision by specifying the crucial influencing mechanism while considering influences from other explanatory processes.

**Figure 1:**
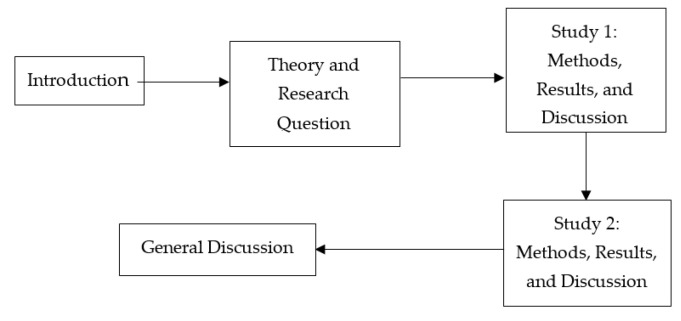
A Flowchart of the Research Process.

Below we first elaborate theory and research methods, which is followed by a successive examination of two separate studies. Finally, we conclude all of our research findings with a general discussion. A flowchart of our research process is illustrated in the below Figure 1.

## 2. Theory and Research Question

### 2.1. Transformational Leadership

Burns [2] and Bass [1] formulated the idea that transformational leadership influences employees to accomplish more than normally expected at work. To unpack the behaviors that a transformational leader may typically display, Bass [1,23] developed a four-dimensional framework, which includes idealized influence, inspirational motivation, intellectual stimulation, and individualized consideration. Podsakoff et al. [24] further enriched this framework by identifying the six subdimensions of transformational leadership behaviors. These are: articulating a compelling vision of the future, providing an appropriate role model, fostering the acceptance of group goals, demonstrating high performance expectations for followers, providing individualized support that considers followers’ feelings and needs, and challenging followers to re-assess assumptions about their work and how it can be performed well.

When transformational leaders exhibit the aforementioned behaviors, they are able to motivate followers to perform various positive behaviors, including sacrificing their self-interests for the sake of organizational goals and performing beyond ordinary expectations [1,6].

### 2.2. Multi-Foci Framework of Transformational Leadership Process

Despite the consensus on what constitutes core transformational leadership behaviors and what outcomes they could induce, our extant knowledge remains limited, particularly regarding the pivotal intermediate process that links transformational leadership to follower outcomes. Thus, we build a framework that includes three foci in the transformational leadership process: “follower dependence [upon the leader]”, “follower independence [from the leader]”, and “leader-follower interdependence”. We include these foci because they represent major approaches through which a transformational leader could influence subordinates. In particular, “follower dependence” refers to a process in which subordinates rely on their leader for guidance and direction because they are impressed at the former’s symbolic power and admire him/her [25]. Nevertheless, the followers’ major motivation in this process is to please the leader. That is, the followers’ independent personalities are not exercised and they are simply executors of their leader’s will [26]. Meanwhile, “follower independence” emphasizes a process in which the subordinates’ abilities and internal motivations are developed. Consequently, the followers’ self-initiatives play a substantial role in doing their jobs well because their independent self-construal is formed and they work to pursue their interests and competence [27]. Lastly, “leader-follower interdependence” depicts a type of alliance that comprises instrumental and emotional bonds between the two parties [17]. This state creates a stable structure for the leader to obtain sustainable good results because leaders and subordinates have reached a tacit understanding of each other’s expectations and have developed mutual obligations and commitment toward each other.

Our theorization and empirical testing below revolve around the three different “foci”. To be consistent, we selected the six-dimensional transformational leadership framework of Podsakoff et al. [24] and its two-resultant performance-beyond-expectation indices (i.e., task performance and OCB) as the starting points to organize our arguments. Our plan is to specify how each “focus” independently translates the effects of transformational leadership upon follower outcomes before discussing how the three foci operate jointly.

### 2.3. Follower Dependence upon the Leader ‘Focus’: The Role of Personal Identification with the Leader

Followers may behave in two different ways to demonstrate their dependence on the leader. On the one hand, they may rely on leaders to proceed with work and make decisions [28]. On the other hand, they tend to develop their work motivation and self-esteem based upon the leader’s recognitions [11]. The current study posits that subordinates become dependent on their transformational leader for both guidance and approval, which is manifested by identifying with their leader.

Shamir et al. [29] stressed the role of identification in transformational leadership processes. Social identity theory indicates that an individual becomes identified with another person when he/she views the essence of that person as self-defining while attaching value and emotional significance to that person [30]. Thus, identification with a leader describes a situation in which individual employees change their sense of self to become what their leader has attempted to socialize them to become. Transformational leaders articulate inspirational visions and goals for the collective good while having high expectations for employee performances [11]. These factors result in employee expansion of their self-concepts, such that they internalize their role-defined identity in the leader–subordinate relationship. Transformational leaders set a personal example [31], provide employees with individualized consideration and support, and encourage employees to re-examine the status quo [24]. These personalized behaviors evoke followers to emulate their leader in terms of beliefs, values, and behaviors. In summary, subordinates develop relational identification with their leader when exposed to transformational leader behaviors.

When employees are strongly identified with their leader, they feel a strong sense of affiliation and show conformity to the latter and are motivated to devote additional effort at work to maintain a high level of self-worth before their leader [11]. Subordinates are willing to spend considerable time and energy on their formal job roles and even undertake additional role behaviors that can benefit their leader (e.g., assisting coworkers, complying with the leader’s requests, and making self-sacrifices and exerting extra effort to please the leader) [32]. Prior research has documented the mediating role that identification with the leader (i.e., personal identification) has on the relationship between transformational leadership and follower performance (e.g., task performance, discretionary voice behavior, and creativity) [33,34].

### 2.4. Follower Independence from the Leader ‘Focus’: The Development of Psychological Empowerment

Personal identification involves a dependence mechanism among subordinates, whereas the independence mechanism involves transformational leadership processes nurturing subordinates’ abilities while triggering their self-initiatives and intrinsic motivations [11].

Transformational leadership may energize followers by enhancing their faith in their ability to accomplish meaningful goals [35]. Such a process can be well captured by the psychological empowerment construct [15], which has four components. These components are meaning (i.e., value of a work goal or purpose in relation to employees’ own standards), competence (i.e., perceived ability to skillfully accomplish work), self-determination (i.e., sense of autonomy in the initiation and continuation of work behaviors), and impact (i.e., degree to which employees can influence work outcomes). First, transformational leaders engage in vision articulation and encourage collective work goal commitment, thereby engendering followers’ seeking meaning from work. Second, transformational leaders strengthen followers’ beliefs that they can accomplish work-related activities by challenging employees to reformulate old problems in new ways while providing support, empathy, and encouragement. Accordingly, followers gain choices in initiating and regulating their own actions. Lastly, transformational leaders set high standards and expect significant performance from their followers, thereby building followers’ beliefs that they make a difference at work.

Empowered employees firmly believe in their capabilities, understand work meanings, and are granted autonomy. Thus, they work due to initiatives and intrinsic motivation for good performance. In addition, empowered employees likely display citizenship behaviors because they feel competent in completing their tasks and they consider assisting their coworkers relevant to their work meaningfulness and effect. Prior studies have drawn on self-determination and intrinsic motivation theories and corroborated that psychological empowerment and core job characteristics, respectively, mediate between transformational leadership and subordinate task performance and OCBs [10,27]. Furthermore, consistent with social learning theory, transformational leadership elicits followers’ beliefs in their ability to perform tasks (i.e., self-efficacy) successfully, thereby affecting their performance, including task performance [36], proactive work behaviors [37], and creativity [38].

### 2.5. Follower Interdependence with the Leader ‘Focus’: Evolution of LMX

Interdependence involves a state of mutual dependence between leaders and followers. Interdependence involves two preconditions. First, both parties have coalitions of interests, such that they share the same goal, or their acts are mutually beneficial [39]. Second, interdependent transactions must be built to provide important resources that are deemed valuable by the other party [40]. As such, this “leader–follower interdependence” focus may include reciprocity between the two parties and interpersonal attachment resulting from a series of interdependent exchanges [41].

High-quality LMX relationships involve interdependence between leaders and followers based on reciprocity and equity. High-quality LMX relationships are achieved through a process of role negotiation and social exchange [16]. Leaders convey role expectations to their followers and provide tangible and intangible resources and rewards to satisfy the followers’ expectations. Moreover, followers hold role expectations for their leaders, particularly with respect to how they are treated and rewarded when they meet their leader’s expectations [42]. This reciprocal process evolves over time, with each party contributing different resources and rewards to satisfy the other’s needs and strengthen the relationship [16]. Transformational leaders may foster the development of high interdependence, which is characteristic of high LMX because transformational leaders articulate inspiring visions, set high performance expectations, and provide subordinates with individualized support. Therefore, followers clearly understand their role responsibilities while simultaneously having the motivation and resources to reciprocate by meeting their leader’s expectations. In addition, other transformational leadership behaviors, such as advocating collective interests and goals and role modeling, can further convince followers that their efforts and contributions to the LMX relationship is worthwhile [43].

After several rounds of reciprocation through mutual expectation fulfillment, high interdependence, characterized by mutual trust, respect and obligation, develop [37]. Thus, task performance is an important form of “currency” in the dyadic social exchange and is likely used by followers to fulfill their reciprocity obligations. In addition, since there is no clear boundary to such obligations [14], OCB, a more discretionary exchange currency, may be used. Accumulated evidence supports LMX as a mediator of the relationship between transformational leadership and task performance and OCB [12,44].

### 2.6. Juxtaposition of the Three Alternative ‘Foci’

After explicating how the three mechanisms or “foci” may separately explain the effects of transformational leadership on individual performance, we incorporate them in one research model and theorize their relative strengths in producing the effects of transformational leadership on subordinate work performance. Such an investigation is worthwhile because it strengthens confidence in prior reported findings and should spotlight the key mechanism(s) underlying transformational leadership effects.

We first go back to transformational leadership theory to seek answers. We identify that leader–member exchange or its underlying leader–follower interdependence “focus” may be the most influential mechanism in the transformational leadership process [2]. Unlike transactional leaders who lead by engaging in economic exchange processes with followers, transformational leaders elevate the leader–follower relationship to a higher level. A quality leader–follower relationship involves mutual liking, respect, trust, and commitment between the two parties and turns “pure” transactions into social exchanges [1].

Alternatively, we argue that followers’ dependence upon the leader can be a pivotal feature of transformational leadership because transformational leadership theory was built upon charismatic leadership theory [45], which stresses personal identification (i.e., how leaders’ charisma molds their follower beliefs, feelings, and behaviors) as the primary influencing mechanism [29,46]. Given that transformational leadership has gone beyond charismatic leadership to include several additional dimensions, such as intellectual stimulation and individualized consideration [24], charisma and role modeling remain as the cornerstones of transformational leadership theory; these later dimensions are simply auxiliaries to assist the “core” dimensions [45].

Lastly, transformational leadership can be argued to be a motivational leadership style that focuses on subordinates’ self-actualization needs [2]. For example, Avolio and Bass [47] indicated that transformational leaders enhance followers’ capability to think on their own and become independent. Similarly, Avolio and Gibbson [48] supported the idea that a major goal of transformational leadership is to facilitate follower development and self-management. These arguments underlie previous scholars’ beliefs that transformational leaders can cultivate employees who think for themselves and are actively engaged in every aspect of their work.

Thus, these arguments suggest that any of the three “foci” can constitute the key mechanism of transformational leadership theory. Thus, we expect that any one of the foci can stand out in the transformational leadership process or all three can be equally important in translating the effects of transformational leadership.

Edwards and Berry [21] have advocated an alternative method of developing comparative hypotheses, which is to draw evidence from the extant empirical studies that specify the primacy of the transformational process. The majority of the studies on transformational leadership have analyzed one mediator at one time (e.g., psychological empowerment as a proxy for the follower independence “focus,” Avolio et al. [49] or multiple mediators from one single perspective (e.g., organizational value internalization and role self-efficacy as two different proxies to reflect the same mechanism, i.e., follower independence [50]). Therefore, we cannot draw clear generalizations from past studies.

In contrast, two recent meta-analyses may be especially informative. Ng [51] identified previous studies that have explored the intermediate mechanisms of transformational leadership on individual performance and confirmed the central role of leader–member exchange in relationships among transformational leadership, other various mediating variables (including job self-efficacy and organizational identification), and performance outcomes. This implies that transformational leadership theory may be an interdependence-based leadership theory. However, Ng’s [51] conclusion is based on sequential mediation. He did not treat several alternative mechanisms as juxtapositions in his computations. Moreover, Gottfredson and Aguinis [7] meta-analyzed 35 prior meta-analyses on leadership influence processes. Leader–member exchange explained the largest variance in performance among various leadership behaviors, including transformational leadership, consideration, and initiating structure. They concluded that relational leadership theory (i.e., leader–member exchange) is the best theoretical explanation of why leadership behaviors (including transformational leadership) lead to follower outcomes. However, because Gottfredson and Aguinis [7] compared leader–member exchange with job satisfaction and commitment as competing mechanisms, we do not know the extent to which their findings are applicable to the current research.

Given that the above perspectives and empirical evidence are rather divergent, and that our three intermediate mechanisms are often correlated with each other, it seems unreasonable to predict which focus is more likely to dominate the transformational leadership process. Thus, we do not propose any specific hypothesis but simply ask a research question to guide the current research:

Research Question: Among follower dependence, independence, and interdependence, which theoretical mechanism dominates the transformational leadership—performance and OCB relationships, or do they work equally well?

## 3. Overview of Present Research

The research question was tested in two studies. The first study used a multi-source, cross-sectional design, while the second employed a multi-source, time-lagged design, in which the independent variable (IV) was measured at Time 1 and the mediators (Ms) and dependent variables (DVs) were measured at Time 2 (to reduce the effects of common method bias [52]).

### 3.1. Study 1: Methods

#### 3.1.1. Participants and Procedures

Survey data were collected from six organizations in Eastern China. We contacted the human resource (HR) management director in each organization to obtain cooperation in the study. Questionnaires (placed in envelopes) were distributed by researchers on sites to a list of supervisors and followers who showed willingness to participate in our study in 2012. The majority of the questionnaires were returned on the same day. For the participants who were unable to complete the survey immediately, follow-up requests were made one week later to guarantee a high response rate.

Surveys were distributed to 300 followers and their direct supervisors (upper- or middle-level managers). To qualify to be retained in the sample, a supervisor must have at least two subordinates who returned the survey to reduce or eliminate potential selection bias. A total of 275 follower and 107 supervisor surveys were returned. After excluding the incomplete or mismatched surveys, a total of 239 valid leader–follower dyads (85 supervisors) were retained.

The average ages of the followers and supervisors were 31.0 years (SD = 7.5) and 35.9 years (SD = 8.0), respectively. The average time for followers under the leadership of their current supervisor was 39 months (SD = 42). A total of 43.5% of the followers were female and 61.1% hold a bachelor’s degree or higher. Among the supervisors, 34.1% were female and 75.7% hold a bachelor’s degree or higher.

#### 3.1.2. Measures

Seven-point Likert scales that range from 1 (Strongly disagree) to 7 (Strongly agree) were used for all the measures except for task performance. All the instruments were translated and back-translated into Chinese to assure item wording validity [53].

*Transformational leadership*. Podsakoff et al.’s [24] 23-item, 6-dimension Transformational Leadership Inventory (TLI) was employed to assess each follower’s perception of their direct supervisor’s transformational leadership (α = 0.94). This study anchored transformational leadership at the individual level because cross-level analysis is often inappropriate for assessing and comparing multiple-mediator models [54].

*Identification with the leader*. Each follower’s identification with their direct supervisor was assessed with five items drawn from the Shamir et al. [31] measure of subordinate identification with the leader (α = 0.87). A sample item is “I am proud to be under his/her command”.

*Psychological empowerment*. Spreitzer’s [15] 12-item measure of psychological empowerment was employed (α = 0.81). A sample item is “I can decide on my own how to go about doing my work.”.

*LMX*. The LMX–MDM scale from Liden and Maslyn [42] was assessed. This scale includes 12 items on 4 dimensions that emphasize mutual liking (i.e., “affect” dimension), faithfulness (i.e., “loyalty” dimension), synergic efforts (i.e., “contribution” dimension), and professional recognition (i.e., “professional respect” dimension) (α = 0.82). A sample item is “I do not mind working my hardest for my supervisor.”.

Task performance was rated by leaders with seven items from Schriesheim et al. [55] on a 5-point Likert scale (5 = excellent, 4 = good, 3 = fair, 2 = not good, and 1 = poor) (α = 0.90). A sample question is “How good would you say is the quality of the performance of this person?”.

*OCB*. We measured OCB using the 24-item, 5-dimension (conscientiousness, sportsmanship, civic virtue, courtesy, and altruism) OCB scale developed by Podsakoff et al. [24] (α = 0.90). Sample items are “willingly helps others who have work related problems” and “attends functions that are not required but helps the company image”.

#### 3.1.3. Analytical Approach

A series of confirmatory factor analyses (CFAs) was first performed to ensure that the operational measures were perceptually distinct from each other. Second, prior to exploring our research question, we determined whether non-independence was a concern because our data contained a hierarchical structure, in which a leader provided performance ratings for at least two followers [56]. The results showed considerable between-level variances in task performance and OCB ratings (intra-class correlation, ICC = 0.22 for task performance and 0.68 for OCB). In addition, one-way analysis of variance (ANOVA) results demonstrated significant between-level differences in task performances (*F* [50, 136] = 1.99, *p* < 0.01) and OCB (*F* [50, 136] = 8.37, *p* < 0.01). Thus, to address the nonindependence concern, we followed Bliese and Hanges [57] in partitioning the variance of our two outcome variables into a within and a between component and constructed a three-mediator model using individual-level transformational leadership to predict within-level task performance and OCB through three concurrent processes. This model was estimated using M-plus 7.4 to obtain point estimations of all the indirect effects and their corresponding standard errors. Given that the M-plus results use symmetric confidence intervals, which assume normality [58], we also employed the Monte Carlo simulation method recommended by Preacher et al. [59] to examine these findings. This additional analysis was undertaken using Selig and Preacher’s [60] web-based utility that generated and ran the R code for the analysis.

### 3.2. Study 1: Results

#### 3.2.1. CFAs

We performed CFAs to assess the psychometric adequacy of the six study variables, namely, transformational leadership (using six subscales as indicators), personal identification, LMX-MDM (using four subscales as indicators), psychological empowerment (using four subscales as indicators), task performance (using three item-construct balanced parcels), and OCB (using the subscales OCB-I and OCB-O as indicators). The resultant six-factor solution, with correlated factors and uncorrelated error, provided a good fit to the data (*χ*2 (215) = 520.56, CFI = 0.91, TLI = 0.89, RMSEA = 0.08, SRMR = 0.06). Then, this model was compared with three alternative models to determine whether the three mediators are perceptually distinct from one another. The top of Table 1 shows that the six-factor solution fits significantly better than the three alternatives, thereby indicating that the three mediators were distinct from one another.

#### 3.2.2. Testing the Research Question

Table 2 (below the diagonal) shows the means, standard deviations, bivariate zero-order correlations, and reliabilities of the Study 1 variables. All scale reliabilities are over 0.80.

The product-of-coefficient method, which yields a symmetric confidence interval [61], was first used to simultaneously estimate the six mediating effects (i.e., three for task performance, and three for OCB). The top of Table 3 shows that only personal identification had a unique mediating effect on the relationship between transformational leadership and OCB (indirect effect = 0.14, 95% CI [0.02, 0.26]) when the other two mediators were present in the model. No mechanism appeared to have a significant unique effect on task performance. Such results indicate that only the personal identification mechanism has an additional contribution beyond LMX and psychological empowerment linking transformational leadership and OCB. In addition, these results show that the total indirect effects through the three mechanisms were significant only for OCB (point estimate = 0.22, 95% CI [0.09, 0.35]). This result suggests that personal identification, LMX, and psychological empowerment jointly (taken as a set) mediated the relationship between transformational leadership and follower OCBs.

Table 4 provides further support to the preceding findings and reports similar results from a parametric bootstrap procedure (with 20,000 Monte Carlo replications) [59]. Of the three theoretical mechanisms, only personal identification emerged as more pertinent in transmitting the effect of transformational leadership to follower OCB (indirect effect = 0.14, 95% bootstrap CI [0.02, 0.27]). Moreover, none of the mediators was shown as pertinent in explaining the relationship between transformational leadership and task performance.

### 3.3. Study 1: Discussion

Personal identification turns out to be the only unique mechanism linking transformational leadership and employee OCB. Thus, this result suggests that the “follower dependence upon the leader” mechanism is stronger than the other two (i.e., “follower independence” and “leader-follower interdependence”) in explaining transformational leadership effects. Furthermore, our results reflect that prior conceptions of transformational effects, such as “lifting ordinary people to extraordinary heights” [62] and “doing more than they are expected to do” [63], seems to apply more to individual employees’ extra-role behaviors rather than to in-role behaviors. Therefore, despite transformational leadership triggering individuals’ multiple psychological mechanisms and inducing various outcomes, follower dependence most strongly influences followers to perform above the scope of their formal duties.

Given that empirical results can be influenced by research context, the generalizability of our findings is a key concern. In particular, one sample characteristic is that all the leaders we surveyed in Study 1 were upper- and middle-level managers. Middle-level managers are responsible for the execution of organizational strategies, whereas upper-level managers generally determine broad strategic strokes for their organizations [64]. We believe that for managers at these two levels, vision articulation and role modeling play especially important roles among the different transformational leader behaviors. Accordingly, the followers’ personal identification may stand out as the dominant influencing mechanism. By contrast, because front-line managers focus on pursuing operational efficiency and performance [65], their individualized consideration and intellectual stimulation behaviors, which are relevant to the psychological mechanisms of leader-member exchange and psychological empowerment [66], may be considerably more important to frontline employees. In summary, our findings may be biased by our research contexts. Therefore, additional research should examine if our results replicate when the sample is composed of frontline employees and their direct supervisors.

Although we used multi-source data to reduce common method bias and our analysis demonstrated that the different constructs were perceptually distinct, concerns about common method bias and causality remain [52]. We also employed the four-dimensional LMX–MDM scale to assess the interdependent mechanism. This may be appropriate at a conceptual level. However, when we analyze the items, this scale is limited in that it emphasizes a one-way directional contribution from subordinates to the leader rather than interdependent exchanges and interpersonal attachment between leader and follower [42].

To address these concerns, we collected a second set of data to replicate our Study 1 results. This sample is composed of frontline workers and their direct supervisors. We also used a time interval between our measurement of the IV and measures of the mediators and DVs. The leader–member social exchange (LMSX) scale [67] was also used to better capture two-way contributions from leaders and followers.

### 3.4. Study 2: Methods

#### 3.4.1. Participants and Procedures

Data were collected from 31 stores of a local state-owned telecommunication company in Southern China. The leading author visited all 31 stores twice in 2013 to conduct the surveys, separated by a one-month interval. The respondents comprised frontline customer service staff and their managers from different stores around the city. Questionnaires were distributed to 148 followers and their direct supervisors. After discarding incomplete, careless, and unmatched surveys, 138 valid leader–follower dyads (from 31 direct supervisors) were retained for further analysis.

The average ages of the followers and supervisors were 30.2 years (SD = 4.6) and 32.5 years (SD = 2.9), respectively. The average time for the followers working for this organization was 82 months (SD = 50). A total of 76.8% of the followers were female and 39.9% hold a bachelor’s degree or higher. Among the supervisors, 80.6% were female and 71% hold a bachelor’s degree or higher.

#### 3.4.2. Measures

All measures, except LMX and OCB, were identical to those employed in Study 1. At time 1, the followers rated their direct supervisor’s transformational leadership behaviors, while the supervisors rated their followers’ task performance and OCB, which were used thereafter as control variables. After one month, the followers provided ratings of their personal identification with the leader, LMX, and psychological empowerment, while the supervisors rated their followers’ task performance and OCB again. This was undertaken to reduce common method bias and improve the study’s design.

*LMX*. Bernerth et al.’s [67] eight-item leader–member social exchange (LMSX) scale was used to assess relational reciprocity and interdependent transactions in the leader–member social exchange relationship; thus, a high score is indicative of high interdependence. LMSX is more content valid than LMX-MDM (used in Study 1) to reflect social exchange relationship [68]. According to Cropanzano and Mitchell [41], social exchange relationship is homogenous to transactional interdependency. Thus, LMSX is an alternative measure to LMX-MDM in terms of its interdependent focus, while superior in terms of psychometric properties. Items of this construct were rated with a seven-point Likert scale (α = 0.93). A sample item is “My relationship with my manager is composed of comparable exchanges of giving and taking.”.

*OCB*. We measured OCB using the 16-item OCB scale developed by Lee and Allen [69] (α = 0.96). Lee and Allen [69] distinguished OCB in terms of the intended beneficiary of the citizenship behavior and measured it with two subscales, namely, OCB toward individuals (OCB-I) (8 items) and OCB toward the organization (OCB-O) (8 items). Sample items for the two subscales are “Help others who have been absent” and “Take actions to protect the organization from potential problems,” respectively.

#### 3.4.3. Analytical Approach

We first analyzed whether non-independence was a concern because the data were obtained from 138 followers nested within 31 leaders [56]. The results showed that between-level variances in both outcome variables (i.e., Time 2 task performance and OCB ratings) were not trivial (intraclass correlation [ICC] = 0.43 for task performance and 0.66 for OCB). ANOVA results also demonstrated that the between-level differences in the two outcome variables were significant for task performance (*F* [30, 109] = 4.75, *p* < 0.01) and for OCB (*F* [30, 109] = 11.70, *p* < 0.01). Thus, the same procedures employed in Study 1 were employed here. In addition, to enhance our specification of the causality [70], we turned our dependent variables from levels to changes by controlling within-level Time 1 task performance to predict Time 2 task performance and within-level Time 1 OCB to predict Time 2 OCB.

### 3.5. Study 2: Results

#### 3.5.1. CFAs

We assessed the factor structure of the six study variables as previously done in Study 1. Three balanced construct item parcels were created for the newly added LMSX measure and the two subscales (OCB-I and OCB-O) were employed for the OCB measure. The six-factor solution provided a good fit to the data (*χ*2 (215) = 373.11, CFI = 0.93, TLI = 0.92, RMSEA = 0.07, SRMR = 0.06), which was compared thereafter with three alternative models. Table 1 (bottom part) shows that the obtained results suggest discriminant validity among the three mediators, thereby permitting research question testing using a multiple mediation approach [54].

#### 3.5.2. Testing the Research Question

Table 2 (above the diagonal) shows the means, standard deviations, bivariate zero-order correlations, and reliabilities of the Study 2 variables. All scale reliabilities are over 0.90.

Table 3 (bottom part) shows that only identification with the leader had a unique mediating effect on the relationship between transformational leadership and OCB (indirect effect = 0.07, 95% CI [0.01, 0.12]) when the other two mediators were present in the model. No mechanism appeared to have a significant effect on task performance, as indicated by all the 95% CIs for the specific indirect effects including zero. In addition, the total indirect effects of transformational leadership on OCB was significant (point estimate = 0.07, 95% CI [0.00, 0.13]). These results were consistent with those that we obtained in the previous study. We further explored these Study 2 findings by analyzing the asymmetric 95% CIs estimates with 20,000 Monte Carlo replications. Table 4 (bottom part) shows that the pattern of the specific indirect effects is again supported.

### 3.6. Study 2: Discussion

Study 2 extends Study 1 in the following two ways. First, we changed the sample from upper- and middle-level managers to frontline managers. Second, we improved the measures and the research designs to reduce common method bias and strengthen the generalizability of our research findings. The fact that Study 2 replicates the results obtained in Study 1 further demonstrates that among the three foci, followers’ identification with the leader (or the “follower dependence” mechanism) is stronger than the other two (i.e., “follower independence” and “leader-follower interdependence”) in translating the effects of transformational leadership on follower OCB.

## 4. Discussion

Transformational leadership has been widely romanticized as one of the “best management practices” [71]. However, as empirical evidence accumulates, our understanding of the essence of this leadership approach remains stagnant. At the conceptual level, this stagnancy may be because “the mediators studied are rather diverse and there is no theory to guide the integration of mediation” [6]. At the empirical level, the majority of the mediators that have been analyzed have been examined in isolation [13]. Van Knippenberg and Sitkin [6] reflected on this current lack of theoretical clarity and empirical integration and noted that “all mediators identified in [transformational leadership] research could plausibly apply equally to all outcomes”.

Using two samples, one with a longitudinal design, our results consistently revealed “follower dependence” (personal identification) as the mechanism that best explains the transformational leadership–follower OCB linkage. In addition, our post-hoc analyses also revealed that each specific mechanism is significant when examined alone. These findings suggest that transformational leadership is perhaps in essence a follower dependence-oriented leadership theory that involves leader facilitating extra-role behaviors via eliciting follower identification. Therefore, we conclude that transformational leadership’s performance-enhancing effects appear to depend upon the abilities of leaders to assimilate followers with their values and visions, rather than to awaken followers’ independence or to develop an interdependent relationship with them.

### 4.1. Theoretical Implications

This study makes several important contributions to the transformational leadership literature and broader leadership theory. First, our primary contribution lies in the integration of three alternative mechanisms, namely, “follower independence,” “follower dependence,” and “follower interdependence with the leader.” The integration of these foci in one study has enabled us to go beyond the traditional single-mediator route to better understand the transformational leadership process. In previous approaches, scholars used one single perspective to model the intermediate process involved in producing transformational effects [4,12,49]. In contrast, our investigation of three seemingly different approaches refines the entire picture and reveals the key process in transformational leadership. Yukl [32] suggested that “the [transformational leadership] theory would be stronger if the essential influence processes were identified more clearly and used to explain how each type of behavior affects each type of mediating variable and outcome”.

This study addresses a long-existing divergence regarding the essence of the transformational leadership process. Prior theory and evidence suggested that any one of the three “foci” could be key in the process. Our research, however, revealed that the transformational leadership process is primarily driven by the follower–dependence mechanism. Thus, if we liken our extant understanding (i.e., there exist diverse routes by which transformational leader behaviors cause follower effects) to “All roads lead to Rome,” then our research refines this understanding by identifying “follower dependence” as the main road and “follower independence” and “follower interdependence with the leader” as two side roads.

This finding is consistent with House and Shamir’s [29] assertion that for transformational leadership theory, the key lies in a leader’s ability to tie a follower’s self-concepts to the actualization of the leader’s goals. Shamir et al. [29] argued that transformational leaders engage follower self-concepts and cause followers to link the valued aspects of their self-concepts to their involvement in the leader’s visions and missions. Nevertheless, this assertion has yet to be adequately tested [12]. Prior evidence shows personal identification (or its represented follower dependence focus) as one important mechanism that links transformational leadership to various outcomes, which includes but are not limited to, voice [33] and creativity [72]. In contrast, our research may be the first to support the primacy of this mechanism in the transformational leadership process. Hence, our research supports House and Shamir’s [29] proposition.

The preceding finding also bears on the debate concerning whether transformational and charismatic leadership are synonymous [29,73,74]. Prior to our study, previous research indicated that follower personal identification and dependence are essential to charismatic leadership [75] and that transformational and charismatic leadership have a large (i.e., 78%) conceptual overlap and achieve a similar relationship with follower performance as rated by the leader [76]. However, we identify another interface between charismatic and transformational leadership by demonstrating that personal identification is central to the transformational leadership process. Thus, our research contributes to this multi-faceted dialogue concerning the extent that these two leadership styles are similar or distinct [77].

Lastly, our research extends the extant leadership process framework by developing a framework that simultaneously includes “follower dependence,” “follower independence,” and “leader–follower interdependence.” This approach is different from previous frameworks that categorized leadership theory into leader- or follower-centric [78] or that classified leadership process in leveraging targets as cognitive, affective, and behavioral [79]. In particular, our approach considers three possible methods by which effective leaders influence their subordinates [78]. That is, the leader can either consolidate his/her control via strengthening follower dependence, emphasizing empowerment by nurturing follower independence, or developing sustainability by creating leader-follower interdependence. In applying this framework to a variety of leadership theories, we realize that certain leadership styles merely stress one method of influence, whereas others exhibit multiple methods of influence. For example, empowering or participative leaders emphasize the “follower independence” focus [80], charismatic or visionary leaders stress the “follower dependence” focus [29,81], and relationship-oriented leadership, such as benevolent leaders, stress the “leader-follower interdependence” focus [82]. Although the focus of “follower dependence” is essential to transformational leadership theory, prior research has indicated that this type of leadership is capable of influencing the three foci. This may explain why transformational leadership goes beyond many contemporary leadership theories in terms of its predictive validity [83].

### 4.2. Limitations and Future Directions

Our research is subject to several concerns that suggest opportunities for future research. One inconsistent empirical finding concerns the differential effects of the follower dependence mechanism on relationships that link transformational leadership to task performance and OCB. The mediating effect of this mechanism was significant in the current research in predicting OCB but not significant in predicting task performance. One plausible reason is that the effects of transformational leadership (i.e., performance beyond expectations) refer more to individuals’ extra- instead of in-role behaviors [24]. Nevertheless, future research must more fully consider the distinctions between different outcomes of interest, and the interrelationship among the three foci in the transformational leadership process.

*Outcomes of interest*. First, the two outcomes are conceptually distinct as having different underlying triggers. For example, the leader’s recognition and guidance, employees’ self-initiatives, and interdependence with the leader can be theoretically important in soliciting job performance. Alternatively, intrinsic motivation at work and interdependent relationships appear to be unnecessary in soliciting follower extra-role behaviors, especially when comparing with personal identification. Given that our research purpose was to reveal the essence of transformational leadership process, we treated task performance and OCB alike in developing our research model. However, the essence of such leadership may depend not only on how transformational leaders behave, but also on which follower outcomes they aim to influence [6]. Therefore, future research should analyze if the transformational leadership process varies depending on specific follower outcomes.

*Interrelationships among different foci*. Another possibility is that the three foci, particularly follower independence and follower dependence, are mutually offsetting in predicting task performance by transformational leadership. Yukl [65] noted that “the more successful the leader is in developing and empowering followers, the less dependent they will be on the leader for future advice and inspiration.” That is, the identification mechanism, which is seen as strengthening subordinates’ dependency upon leaders, seems incompatible with the self-determinant nature of the psychological empowerment mechanism in soliciting job performance. Nevertheless, Kark et al. [11] found that transformational leadership could simultaneously trigger the mechanisms of follower independence (i.e., psychological empowerment) and follower dependence (i.e., personal identification). Therefore, a meaningful future question is to study how these two or three elements can be simultaneously activated by a transformational leader.

*Cultural generalizability*. Our two studies employed samples from one country (China) that is characterized by high power distance and collectivism. People from such a context attach more value to economic security and relational affiliation than to self-worth and intrinsic motivation [84]. Thus, our research findings may be, at least in part, culturally specific. We recommend that future studies replicate our studies in a Western context to address this concern. Prior research has validated the transformational leadership construct in different cultural contexts and documented that the overarching influencing mechanisms of transformational leadership are the same in Western and Chinese societies [85]; however, “there may be notable differences in the manifestations of these mechanisms” [86] that have to be identified and understood.

*Time frame*. Our study assumed that transformational leadership could simultaneously elicit three divergent psychological mechanisms in followers. What we did not consider was the time frame in terms of when each specific mechanism is engaged. Various follower mechanisms may have emerged at different times when interacting with a transformational boss. For example, common wisdom has suggested that only when an individual becomes completely independent could he/she develop truly interdependent bonds with other people [87]. Hence, only when followers become independent (i.e., they are self-motivated, competent, and psychologically self-sufficient) would they develop truly interdependent relationships with their leader. Alternatively, self-concept based theory [29] posits that followers internalize their leader’s values and beliefs into their self-concepts, such that their behaviors become products of self-consistency and self-congruence, a process much like the follower dependence which emerges ahead of follower independence. Hence, transformational leadership may exert its effects through multiple stages over time [36,51]. If possible, future research should measure various mechanisms simultaneously across different time points and compare different sequential routes to elucidate how the transformational leadership process unfolds in time to influence followers.

*Other leadership theories*. Similar in some ways to transformational leadership, ethical [88], servant [89], and authentic [90] leadership theories also exist. When Brown et al. [88] coined the concept of ethical leadership, they drew heavily from social learning theory and proposed that the nurturing of followers’ independent reasoning on morality is essential to this type of leadership, thereby making it similar to follower independence-oriented leadership theory. However, additional development [20] has extended the process of ethical leadership to include various intermediate mechanisms, such as follower dependence and leader–follower interdependence. The same situation likewise exists with respect to servant leadership, which was originally hypothesized as a leadership style that involved serving others and bringing out the best in followers [89]. However, this leadership style has also been documented to trigger several types of intermediate processes. We can also say essentially the same about authentic leadership. Thus, when possible, future researchers could consider applying our framework to these newly emerged leadership approaches.

## 5. Conclusions

Ever since Burns [2] proposed this new genre of leadership called transformational leadership 40 years ago, a large amount of scholarly work has been conducted to investigate how various mechanisms separately mediate between transformational leadership and follower outcomes. Moving beyond the extant understanding that transformational leadership is an omnipotent leadership theory that leads to “performance beyond expectations” through facilitating various individual’s psychological mechanisms (“follower dependence”, “follower independence”, and “follower interdependence with the leader”), our current research has suggested that transformational leadership does so primarily via a “follower-dependence” mechanism.

## Figures and Tables

**Table 1:** Results of the Measurement Model Confirmatory Factor Analyses.

	*df*	*χ*2	*CFI*	*TLI*	*RMSEA*	*SRMR*
**Study 1:**						
6-factor model	215	520.56	0.91	0.89	0.08	0.06
5-factor model (identification and LMX collapsed to form one factor)	220	558.21	0.90	0.89	0.08	0.06
5-factor model (identification and psychological empowerment collapsed to form one factor)	220	630.80	0.88	0.86	0.09	0.06
5-factor model (LMX and psychological empowerment collapsed to form one factor)	220	607.40	0.89	0.87	0.09	0.06
**Study 2:**						
6-factor model	215	373.11	0.93	0.92	0.07	0.06
5-factor model (identification and LMX collapsed to form one factor)	220	619.42	0.83	0.81	0.11	0.07
5-factor model (identification and psychological empowerment collapsed to form one factor)	220	492.70	0.89	0.87	0.09	0.07
5-factor model (LMX and psychological empowerment collapsed to form one factor)	220	547.09	0.86	0.84	0.10	0.10

**Table 2:** Variable Means, Standard Deviations, Correlations, and Reliabilities.

Variable	M	SD	α a	1	2	3	4	5	6
M				5.55	5.82	5.04	5.42	3.38	5.47
SD				1.02	0.95	1.11	0.82	0.74	0.99
α a				0.97	0.90	0.93	0.92	0.93	0.96
1. TFL b	5.70	0.71	0.94		0.58 **	0.42 **	0.28 **	0.21 *	0.26 **
2. Personal identification	5.81	0.83	0.87	0.73 **		0.56 **	0.45 **	0.36 **	0.41 **
3. LMX c	5.86	0.83	0.82	0.75 **	0.75 **		0.29 **	0.26 **	0.29 **
4. Psychological empowerment	5.44	0.72	0.81	0.40 **	0.46 **	0.48 **		0.18 *	0.11
5. Task performance	3.51	0.63	0.90	0.15 *	0.17 **	0.16 *	0.20 **		0.70 **
6. OCB d	5.59	0.62	0.90	0.12	0.22 **	0.16 *	0.13 *	0.59 **	

a α = Cronbach’s coefficient α, internal consistency reliability; b TFL = Transformational Leadership; c LMX = Leader–Member Exchange; d OCB = Organizational Citizenship Behavior; * *p* < 0.05; ** *p* < 0.01.

**Table 3:** Multiple Mediating Effects of Transformational Leadership on Employee Performance, Symmetric (Assumed Normal) Confidence Intervals.

	Task Performance			OCB		
		95% CI			95% CI	
	Point Estimate	Lower	Upper	Point Estimate	Lower	Upper
**Study 1 ^a^**						
	**Indirect effects**			**Indirect effects**		
Personal identification	0.02	−0.11	0.15	0.14	0.02	0.26
LMX	0.06	−0.04	0.16	0.08	−0.01	0.17
Psychological empowerment	0.04	−0.03	0.10	−0.00	−0.06	0.06
*Total indirect effects*	*0.12*	*−0.01*	*0.25*	*0.22*	*0.09*	*0.35*
**Study 2 ^b^**						
	**Indirect effects**			**Indirect effects**		
Personal identification	0.05	−0.01	0.10	0.07	0.02	0.12
LMX	0.01	−0.02	0.04	0.01	−0.03	0.05
Psychological empowerment	−0.01	−0.04	0.02	−0.01	−0.04	0.02
*Total indirect effects*	*0.05*	*−0.00*	*0.10*	*0.07*	*0.00*	*0.13*

^a^*n* = 239 nested in 85 groups; ^b^ *n* = 138 nested in 31 groups.

**Table 4:** Multiple Mediating Effects of Transformational Leadership on Employee Performance, Asymmetric (Monte Carlo) Confidence Intervals.

	Task Performance			OCB		
		Monte Carlo 95% CI			Monte Carlo 95% CI	
	Point Estimate	Lower	Upper	Point Estimate	Lower	Upper
**Study 1 ^a^**						
	Indirect effects			Indirect effects		
Personal identification	0.02	−0.01	0.16	0.14	0.02	0.27
LMX	0.06	−0.03	0.16	0.08	−0.01	0.18
Psychological empowerment	0.04	−0.02	0.11	−0.00	−0.06	0.06
**Study 2 ^b^**						
	Indirect effects			Indirect effects		
Personal identification	0.05	−0.01	0.10	0.07	0.01	0.12
LMX	0.01	−0.02	0.04	0.01	−0.03	0.05
Psychological empowerment	−0.01	−0.04	0.03	−0.01	−0.05	0.02

^a^*n* = 239 nested in 85 groups; ^b^ *n* = 138 nested in 31 groups.

## References

[1] Bass B.M. (1985). Leadership and Performance beyond Expectations.

[2] Burns J.M. (1978). Leadership.

[3] Shin S.J., Zhou J. (2003). Transformational leadership, conservation, and creativity: Evidence from Korea. Acad. Manag. J..

[4] Kirkman B.L., Chen G., Farh J.-L., Chen Z.X., Lowe K.B. (2009). Individual power distance orientation and follower reactions to transformational leaders: A cross-level, cross-cultural examination. Acad. Manag. J..

[5] Triana M.D.C., Richard O.C., Yücel I. (2017). Status incongruence and supervisor gender as moderators of the transformational leadership to subordinate affective organizational commitment relationship. Pers. Psychol..

[6] Van Knippenberg D., Sitkin S.B. (2013). A critical assessment of charismatic-transformational leadership research: Back to the drawing board?. Acad. Manag. Ann..

[7] Gottfredson R.K., Aguinis H. (2017). Leadership behaviors and follower performance: Deductive and inductive examination of theoretical rationales and underlying mechanisms. J. Organ. Behav..

[8] Huang X., Iun J., Liu A., Gong Y. (2010). Does participative leadership enhance work performance by inducing empowerment or trust? The differential effects on managerial and non-managerial subordinates. J. Organ. Behav..

[9] Zhang X., Bartol K.M. (2010). Linking empowering leadership and employee creativity: The influence of psychological empowerment, intrinsic motivation, and creative process engagement. Acad. Manag. J..

[10] Dust S.B., Resick C.J., Mawritz M.B. (2014). Transformational leadership, psychological empowerment, and the moderating role of mechanistic-organic contexts. J. Organ. Behav..

[11] Kark R., Shamir B., Chen G. (2003). The two faces of transformational leadership: Empowerment and dependency. J. Appl. Psychol..

[12] Wang H., Law K.S., Hackett R.D., Wang D., Chen Z. (2005). Leader-member exchange as a mediator of the relationship between transformational leadership and followers’ performance and organizational citizenship behavior. Acad. Manag. J..

[13] Bono J.E., McNamara G. (2011). From the editors: Publishing in “AMJ”—Part 2: Research design. Acad. Manag. J..

[14] Blau P.M. (1964). Exchange and Power in Social Life.

[15] Spreitzer G.M. (1995). Psychological empowerment in the workplace: Dimensions, measurement, and validation. Acad. Manag. J..

[16] Graen G.B., Uhl-Bien M. (1995). Relationship-based approach to leadership: Development of leader-member exchange (LMX) theory of leadership over 25 years: Applying a multi-level multi-domain perspective. Leadersh. Q..

[17] Schriesheim C.A., Castro S.L., Cogliser C.C. (1999). Leader-member exchange (LMX) research: A comprehensive review of theory, measurement, and data-analytic practices. Leadersh. Q..

[18] Wang G., Oh I.-S., Courtright S.H., Colbert A.E. (2011). Transformational leadership and performance across criteria and levels: A meta-analytic review of 25 years of research. Group Organ. Manag..

[19] Aryee S., Walumbwa F.O., Zhou Q., Hartnell C.A. (2011). Transformational leadership, innovative behavior, and task performance: Test of mediation and moderation processes. Hum. Perform..

[20] Walumbwa F.O., Meyer D.M., Wang P., Wang H., Workman K., Christensen A.L. (2011). Linking ethical leadership to employee performance: The roles of leader–member exchange, self-efficacy, and organizational identification. Organ. Behav. Hum. Decis. Process..

[21] Edwards J.R., Berry J.W. (2010). The presence of something or the absence of nothing: Increasing theoretical precision in management research. Organ. Res. Methods.

[22] Van Der Ven A.H. (2007). Engaged Scholarship: A Guide for Organizational and Social Research.

[23] Bass B.M. (1999). Two decades of research and development in transformational leadership. Eur. J. Work Organ. Psychol..

[24] Podsakoff P.M., MacKenzie S.B., Moorman R.H., Fetter R. (1990). Transformational leader behaviors and their effects on followers’ trust in leader, satisfaction, and organizational citizenship behaviors. Leadersh. Q..

[25] Conger J.A., Kanungo R.N. (1987). Toward a behavioral theory of charismatic leadership in organizational settings. Acad. Manag. Rev..

[26] Barbuto J.E. (1997). Taking the charisma out of transformational leadership. J. Soc. Behav. Personal..

[27] Piccolo R.F., Colquitt J.A. (2006). Transformational leadership and job behaviors: The mediating role of core job characteristics. Acad. Manag. J..

[28] Howell J.M., Conger J.A., Kanungo R.N. (1988). Two faces of charisma: Socialized and personalized leadership in organizations. Charismatic Leadership.

[29] Shamir B., House R.J., Arthur M.B. (1993). The motivational effects of charismatic leadership: A self-concept based theory. Organ. Sci..

[30] Tajfel H. (1978). Differentiation between Social Groups: Studies in the Social Psychology of Intergroup Relations.

[31] Shamir B., Zakay E., Breinin E., Popper M. (1998). Correlates of charismatic leader behavior in military units: Subordinates’ attitudes, unit characteristics, and superiors’ appraisals of leader performance. Acad. Manag. J..

[32] Yukl G. (1999). An evaluation of conceptual weakness in transformational and charismatic leadership theories. Leadersh. Q..

[33] Liu W., Zhu R., Yang Y. (2010). I warn you because I like you: Voice behavior, employee identifications, and transformational leadership. Leadersh. Q..

[34] Wang X.-H.F., Howell J.M. (2012). A multilevel study of transformational leadership, identification, and follower outcomes. Leadersh. Q..

[35] Bennis W.G., Nanus B. (1985). Leaders.

[36] Walumbwa F.O., Hartnell C.A. (2011). Understanding transformational leadership-employee performance links: The role of relational identification and self-efficacy. J. Occup. Organ. Psychol..

[37] Den Hartog D.N., Belschak F.D. (2012). When does transformational leadership enhance employee proactive behavior? The role of autonomy and role breadth self-efficacy. J. Appl. Psychol..

[38] Gong Y., Huang J.-C., Farh J.-L. (2009). Employee learning orientation, transformational leadership, and employee creativity: The mediating role of employee creative self-efficacy. Acad. Manag. J..

[39] Van Lange P.A.M., Rusbult C.E., Van Lange P.A.M., Kruglanski A.W., Higgins E.T. (2012). Interdependence theory. Handbook of Theories of Social Psychology.

[40] Kelley H.H., Thibaut J.W. (1978). Interpersonal Relations: A Theory of Interdependence.

[41] Cropanzano R., Mitchell M.S. (2005). Social exchange theory: An interdisciplinary review. J. Manag..

[42] Liden R.C., Maslyn J.M. (1998). Multidimensionality of leader-member exchange: An empirical assessment through scale development. J. Manag..

[43] Boer D., Deinert A., Homan A.C., Voelpel S.C. (2016). Revisiting the mediating role of leader–member exchange in transformational leadership: The differential impact model. Eur. J. Work Organ. Psychol..

[44] Dulebohn J.H., Bommer W.H., Liden R.C., Brouer R.L., Ferris G.R. (2012). A meta-analysis of antecedents and consequences of leader-member exchange: Integrating the past with an eye toward the future. J. Manag..

[45] House R.J.A., Hunt J.G., Larson L.L. (1977). 1976 theory of charismatic leadership. Leadership: The Cutting Edge.

[46] Bass B.M. (1990). Bass and Stogdill’s Handbook of Leadership.

[47] Avolio B.J., Bass B.M. (1998). You can drag a horse to water but you can’t make it drink unless it is thirsty. J. Leadersh. Stud..

[48] Avolio B.J., Gibbons T.C., Conger J.A., Kanungo R.N. (1998). Developing transformational leaders: A life span approach. The Jossey-Bass Management Series. Charismatic Leadership: The Elusive Factor in Organizational Effectiveness.

[49] Avolio B.J., Zhu W.C., Koh W., Bhatia P. (2004). Transformational leadership and organizational commitment: Mediating role of psychological empowerment and moderating role of structural distance. J. Organ. Behav..

[50] Hannah S.T., Schaubroeck J.M., Peng A.C. (2016). Transforming followers’ value internalization and role self-efficacy: Dual processes promoting performance and peer norm-enforcement. J. Appl. Psychol..

[51] Ng T.W.H. (2017). Transformational leadership and performance outcomes: Analyses of multiple mediation pathways. Leadersh. Q..

[52] Podsakoff P.M., MacKenzie S.C., Lee J.-Y., Podsakoff N.P. (2003). Common method biases in behavioral research: A critical review of the literature and recommended remedies. J. Appl. Psychol..

[53] Brislin R., Lonner W.J., Berry J.W. (1986). The wording and translation of research instruments. Field Methods in Cross-Cultural Research.

[54] Preacher K.J., Hayes A.F. (2008). Asymptotic and resampling strategies for assessing and comparing indirect effects in multiple mediator models. Behav. Res. Methods.

[55] Schriesheim C.A., Neider L.L., Scandura T.A. (1998). Delegation and leader-member exchange: Main effects, moderators, and measurement issues. Acad. Manag. J..

[56] Bliese P.D., Klein K.J., Katherine S.W.J. (2000). Within-group agreement, non-independence, and reliability: Implications for data aggregation and analysis. Multilevel Theory, Research, and Methods in Organizations: Foundations, Extensions, and New Directions.

[57] Bliese P.D., Hanges P.J. (2004). Being both too liberal and too conservative: The perils of treating grouped data as though they were independent. Organ. Res. Methods.

[58] MacKinnon D.P., Lockwood C.M., Williams J. (2004). Confidence limits for the indirect effect: Distribution of the product and resampling methods. Multivar. Behav. Res..

[59] Preacher K.J., Zyphur M.J., Zhang Z. (2010). A general multilevel SEM framework for assessing multilevel mediation. Psychol. Methods.

[60] Selig J.P., Preacher K.J. Monte Carlo Method for Assessing Mediation: An Interactive Tool for Creating Confidence Intervals for Indirect Effects [Computer Software] 2008. http://www.quantpsy.org.

[61] Sobel M.E. (1982). Asymptotic confidence intervals for indirect effects in structural equation models. Sociol. Methodol..

[62] Boal K.B., Bryson J.M., Hunt J.G., Baliga B.R., Dachler H.P., Schriesheim C.A. (1998). Charismatic leadership: A phenomenological and structural approach. International Leadership Symposia Series: Emerging Leadership Vistas.

[63] Yukl G. (1989). Managerial leadership: A review of theory and research. J. Manag..

[64] Floyd S., Wooldridge B. (1994). Dinosaurs or dynamos? Recognizing middle management’s strategic roles. Acad. Manag. Perspect..

[65] Purcell J., Hutchinson S. (2007). Front-line managers as agents in the HRM-performance causal chain: Theory, analysis and evidence. Hum. Resour. Manag. J..

[66] Zacher H., Pearce L.K., Rooney D., McKenna B. (2014). Leaders’ personal wisdom and leader–member exchange quality: The role of individualized consideration. J. Bus. Ethics.

[67] Bernerth J.B., Armenakis A.A., Field H.S., Giles W.F., Walker H.J. (2007). Leader–member social exchange (LMSX): Development and validation of a scale. J. Organ. Behav..

[68] Colquitt J.A., Baer M.D., Long D.M., Halvorsen-Ganepola M.D.K. (2014). Scale indicators of social exchange relationships: A comparison of relative content validity. J. Appl. Psychol..

[69] Lee K., Allen N.J. (2002). Organizational citizenship behavior and workplace deviance: The role of affect and cognitions. J. Appl. Psychol..

[70] Antonakis J., Bendahan S., Jacquart P., Lalive R. (2010). On making causal claims: A review and recommendations. Leadersh. Q..

[71] Tsui A.S., Wang H., Xin K., Zhang L.H., Fu P.P. (2004). Let a thousand flowers bloom: Variation of leadership styles among Chinese CEOs. Organ. Dyn..

[72] Qu R., Janssen O., Shi K. (2015). Transformational leadership and follower creativity: The mediating role of follower relational identification and the moderating role of leader creativity expectations. Leadersh. Q..

[73] Den Hartog D.N., House R.J., Hanges P.J., Ruiz-Quintanilla S.A., Dorfman P.W., Abdalla I.A., Adetoun B.S., Aditya R.N., Agourram H., Akande A. (1999). Culture specific and cross-culturally generalizable implicit leadership theories: Are attributes of charismatic/transformational leadership universally endorsed?. Leadersh. Q..

[74] House R.J., Shamir B., Chemers M.M., Ayman R. (1993). Towards the integration of transformational, charismatic, and visionary theories. Leadership Theory and Research: Perspectives and Directions.

[75] Spencer M.E. (1973). What is Charisma?. Br. J. Sociol..

[76] Rowold J., Heinitz K. (2007). Transformational and charismatic leadership: Assessing the convergent, divergent and criterion validity of the MLQ and the CKS. Leadersh. Q..

[77] Avolio B.J., Yammarino F.J., Avolio B.J., Yammarino F.J. (2013). Introduction to, and overview of, transformational and charismatic leadership. Transformational and Charismatic Leadership: The Road Ahead.

[78] Howell J.M., Shamir B. (2005). The role of followers in the charismatic leadership process: Relationships and their consequences. Acad. Manag. Rev..

[79] Fischer T., Dietz J., Antonakis J. (2017). Leadership process models: A review and synthesis. J. Manag..

[80] Lorinkova N.M., Pearsall M.J., Sims H.P. (2013). Examining the differential longitudinal performance of directive versus empowering leadership in teams. Acad. Manag. J..

[81] Schweitzer A. (1974). Theory and Political Charisma. Comp. Stud. Soc. Hist..

[82] Chan S.C.H., Mak W. (2012). Benevolent leadership and follower performance: The mediating role of leader–member exchange (LMX). Asia Pac. J. Manag..

[83] Hoch J.E., Bommer W.H., Dulebohn J.H., Wu D. (2018). Do ethical, authentic, and servant leadership explain variance above and beyond transformational leadership? A meta-analysis. J. Manag..

[84] Hofstede G.H. (1991). Cultures and Organizations: Software of the Mind.

[85] Dulebohn J., Wu D., Liao C., Hoch J.E. (2017). Transformational leadership and national culture: A meta-analysis across 36 nations. Acad. Manag. Proc..

[86] Huang X., Huang X., Bond M. (2012). The romance of motivational leadership: How do Chinese leaders motivate employees?. Handbook of Chinese Organizational Behavior: Integrating Theory, Research and Practice.

[87] Covey S.R. (1989). The 7 Habits of Highly Effective People: Powerful Lessons in Personal Change.

[88] Brown M.E., Treviño L.K., Harrison D.A. (2005). Ethical leadership: A social learning perspective for construct development and testing. Organ. Behav. Hum. Decis. Process..

[89] Liden R.C., Wayne S.J., Zhao H., Henderson D. (2008). Servant leadership: Development of a multidimensional measure and multi-level assessment. Leadersh. Q..

[90] Avolio B.J., Gardner W.L. (2005). Authentic leadership development: Getting to the root of positive forms of leadership. Leadersh. Q..

